# Critical Case of a Preterm Infant Infected With Respiratory Syncytial Virus Managed in the Pediatric Intensive Care Unit: A Case Report

**DOI:** 10.7759/cureus.52119

**Published:** 2024-01-11

**Authors:** Amaal F Alshihabi, Saleh A Alnass, Fatimah S Alsammak, Muhammad S Al Abdrabalnabi

**Affiliations:** 1 Pediatric Intensive Care Unit, Jubail General Hospital, Jubail, SAU; 2 Respiratory Medicine, Jubail General Hospital, Jubail, SAU; 3 Nursing, Qatif Central Hospital, Qatif, SAU; 4 Respiratory Therapy, Jubail General Hospital, Jubail, SAU

**Keywords:** non-invasive positive pressure ventilation (nippv), high-frequency oscillator ventilator (hfov), positive end-expiratory pressure (peep), pressure control ventilation method, respiratory tract, high flow nasal cannula (hfnc), pediatric intensive care unit(picu), acute bronchiolitis, inhaled nitric oxide, respiratory syncytial virus (rsv)

## Abstract

We describe a critical case of a respiratory syncytial virus (RSV) infection in a preterm infant resulting in respiratory failure with advanced respiratory interventions and discharge from our hospital without the requirement for home oxygen therapy or tube feeding. The infant, delivered at 35 weeks gestation due to a premature rupture of the membranes with a birth weight of 2 kg, initially demonstrated a stable postnatal course. The baby required no resuscitation, with Apgar scores of 8 and 9 at one and five minutes, respectively. The infant was discharged in good condition after four days of hospitalization. This report presents a critical case of RSV infection in a preterm infant requiring intensive care. The infant, born at 35 weeks gestation, initially appeared healthy but developed severe symptoms at 22 days old. The emergency evaluation revealed significant respiratory distress and confirmed RSV pneumonia. Following extensive interventions, including mechanical ventilation to manage severe symptoms, along with complications such as pneumothorax and a cardiac arrest episode, the infant exhibited a positive response to subsequent treatments. The infant responded positively to high-frequency oscillatory ventilation and inhaled nitric oxide. Gradual weaning from advanced ventilation led to successful extubation, followed by recovery with high-flow nasal cannula therapy. The case highlights the challenges of managing severe RSV infections in preterm infants and the efficacy of intensive care interventions in facilitating the infant's remarkable recovery and discharge.

## Introduction

The respiratory syncytial virus (RSV) is a common virus that primarily causes mild, cold-like symptoms but can lead to severe respiratory illness, especially in infants and elderly adults. It is a member of the Paramyxoviridae family and is characterized by symptoms such as coughing, sneezing, runny nose, fever, and, in more severe cases, bronchiolitis and pneumonia. Highly contagious RSV spreads via respiratory droplets and can survive on surfaces, posing a significant risk to young children (especially premature infants) [1], older adults, and individuals with weakened immune systems or chronic heart and lung diseases. Diagnosis is typically based on clinical signs and confirmed through tests such as nasal swabs. Although there is no specific treatment for RSV, management involves supportive care such as hydration and fever reducers. Severe cases require hospitalization for oxygen therapy or ventilation. Preventative measures include good hygiene practices, avoidance of contact with sick individuals, and surface disinfection. RSV infections generally peak during the fall, winter, and spring, vary by location, and make awareness and prevention strategies critical during these seasons.

Background

RSV is the major cause of lower respiratory tract illness in children. For most children, an initial RSV infection normally occurs within the first two years of life. In infants less than one year of age and with lower respiratory infections, up to 80% are caused by RSV. In most cases, the virus is not fatal [1]. The most severe infections occur in well-defined high-risk groups that include infants with a history of premature birth and those with chronic lung disease, congenital heart disease, cystic fibrosis, and immunodeficiency [1-2]. However, most children with RSV infections were previously healthy, and it is often difficult to predict the deterioration of an RSV infection.

## Case presentation

Clinical presentation

A male infant was born to a 26-year-old mother with no significant medical history. The infant, delivered at 35 weeks gestation due to a premature rupture of the membranes with a birth weight of 2 kg, initially demonstrated a stable postnatal course and was discharged in good condition after four days of hospitalization. At 22 days of age, the infant presented with a cough and poor feeding for two days. Per the mother’s report, he began to exhibit severe cough and cyanosis abruptly without prior fever, diarrhea, convulsive episodes, abnormal posturing, or skin rashes. The family reported a history of upper respiratory tract infections with fever.

Emergency room evaluation

**Figure 1:**
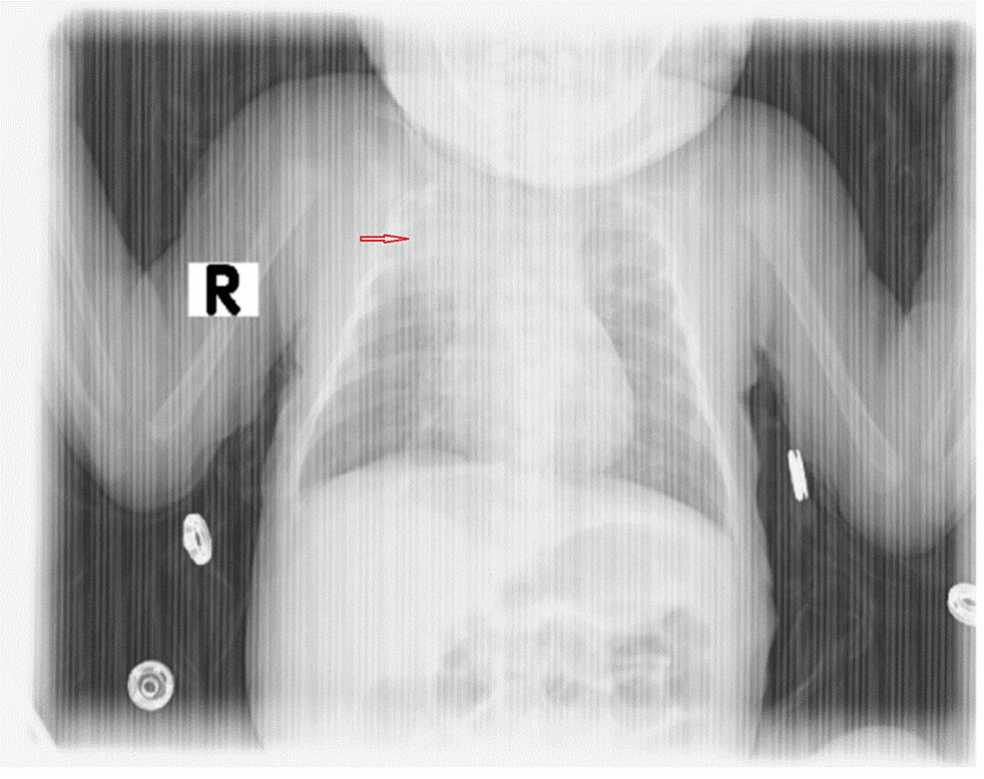
Chest X-rays with the right upper lobe collapse This is a chest radiograph showing a visible opacity in the upper right lung field, indicative of a dense, collapsed area with reduced volume compared to surrounding lung regions.

Upon presentation to the emergency room, the patient was lethargic and pallid and showed moderate respiratory distress, retraction, and grunting. Vital signs were notable for a heart rate of 120 bpm, blood pressure of 110/70 mmHg, respiratory rate of 68 breaths per minute, and oxygen saturation of 99% on a 4 L/min nasal cannula. Physical examination revealed a weight of 2.2 kg, a height of 32 cm, decreased air entry on the right side of the chest with bilateral crepitation on auscultation, a soft abdomen without organomegaly, and no audible cardiac murmurs (notable for loud crepitus). A chest x-ray indicates right upper lobe collapse (Figure 1).

Intensive care and clinical course

The child was brought to the pediatric intensive care unit (PICU) [3] for persistent observation and therapy involving regular nebulization, chest physiotherapy, and nasal suctioning. Unfortunately, the child’s health worsened, necessitating the start of continuous positive airway pressure (CPAP: 10-12 cm H2O) therapy, which was not well received for two days. Following this, he required intubation and mechanical ventilation support due to severe hypoxemia and severe respiratory distress [4].

In spite of receiving the highest level of ventilator support (on pressure control ventilation mode with settings of pressure control (PC: 24 cm H2O), respiratory rate per minute (RR: 50 breaths per minute), positive end-expiratory pressure (PEEP: 6 cm H2O), and the fraction of inspired oxygen (FiO2: 100%), the patient’s condition continued to decline over a two-week period. During this time, the child's health experienced fluctuations but stabilized. Monitored the infant's condition through daily laboratory tests, chest X-rays, and vital sign assessments. On admission, he had been started on ampicillin 200 mg/kg/day and cefotaxime 150/kg/day. His condition worsened, a blood investigation workup was done (Table 1), and his antibiotics were updated to meropenem and vancomycin, azithromycin, and Tamiflu. tracheal-aspirate specimen polymerase chain reaction confirmed RSV pneumonia.

**Table 1:** Laboratory findings upon admission Hgb: hemoglobin; WBC: white blood cell; Na+: sodium; K+: potassium; BUN: blood urea nitrogen; PT: prothrombin time; PTT: partial thromboplastin time; INR: international normalized ratio.

Laboratory investigation	Upon admission
Hgb	12.0 g/dL
WBC	21.00 × 10^3^/µL
Platelets	127 × 10^3^/µL
Na^+^	146 mmol/l
K^+^	3.1 mmol/l
BUN	5
Creatinine	33
PT	15 s
PTT	51.4 s
INR	1.2
Blood culture	Negative

**Figure 2:**
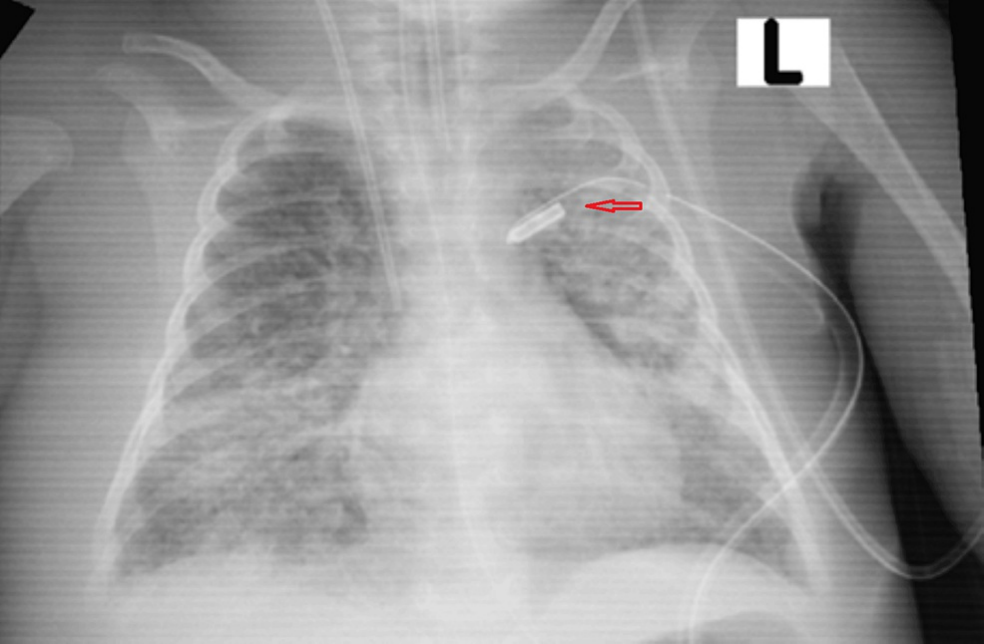
Chest X-rays with tube insertion This is a post-interventional chest radiograph, following intercostal tube (ICT) placement, demonstrating the re-expansion of the previously collapsed lung. However, the remaining extensive pulmonary infiltrate is indicative of persistent parenchymal disease.

**Figure 3:**
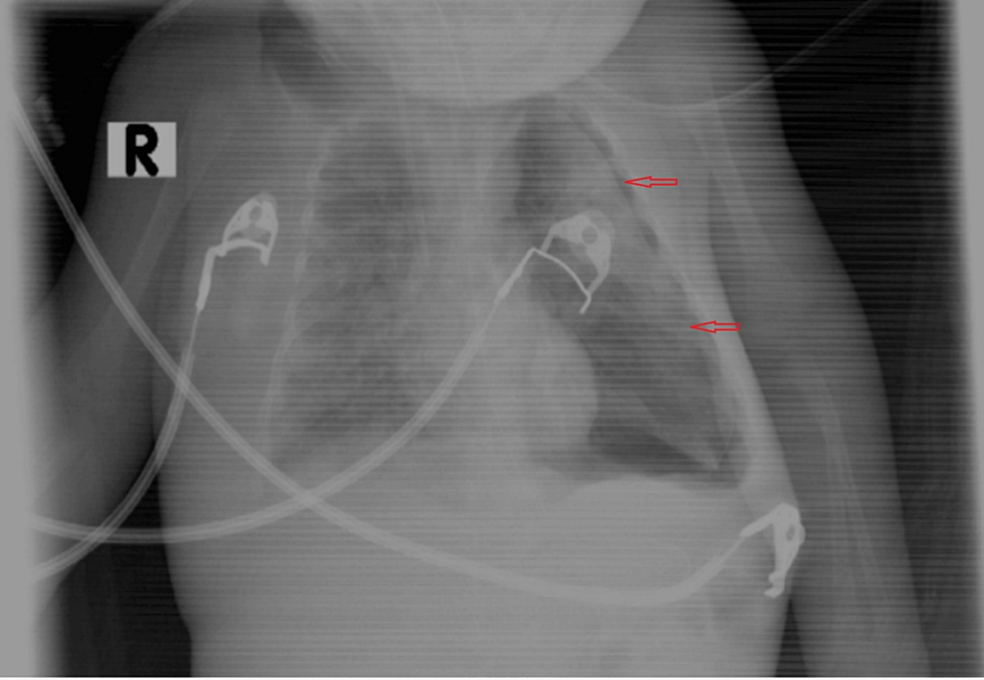
Chest X-rays with the presence of a tension pneumothorax on the left side This is a chest radiograph indicating the presence of a tension pneumothorax on the left side, characterized by the displacement of mediastinal structures away from the affected side, along with the collapse of the left lung and a significant pressure effect on the thoracic components.

The child experienced varying oxygen saturation levels ranging from 82 to 91%, and blood gas analysis indicated ongoing acidosis and hypoxia. Additionally, the patient developed a left-sided pneumothorax due to elevated ventilator settings (Figure 2), initially treated with needle decompression and then with the placement of an intercostal tube (Figure 3).

Because of the severity of his condition and severe desaturation (spo2 50-60%), he developed cardiac arrest for four minutes, which was resuscitated well by chest compression and medication (epinephrine 0.022 mg/kg). After that, the patient was switched to high-frequency oscillatory ventilation (HFOV) and started on 40 PPM of inhaled nitric oxide (iNO). This combination of treatments resulted in a notable improvement in his clinical condition and oxygenation levels [5-6]. An echocardiogram conducted during this period showed no signs of pulmonary hypertension. At this point, the patient was in a very bad condition as well as on inotropic support with dopamine up to 15 mic/kg/min, dobutamine 7.5 mic/kg/min, and noradrenaline 0.7 mic/kg/min. Also, the antibiotic fluconazole was given due to thrombocytopenia (blood culture for candida negative) and other species from the central lines of both lumens.

The patient was maintained on HFOV for five days, reaching high settings (HZ 6, amplitude 45, MAP 20, FiO2 100) with a maximum iNO of 55 PPM. The total duration of iNO treatment was five days. As his condition began to improve [5-6], the medical team gradually reduced these settings and transitioned him back to a conventional ventilator for three days before successfully extubating him.

Recovery and discharge

**Figure 4:**
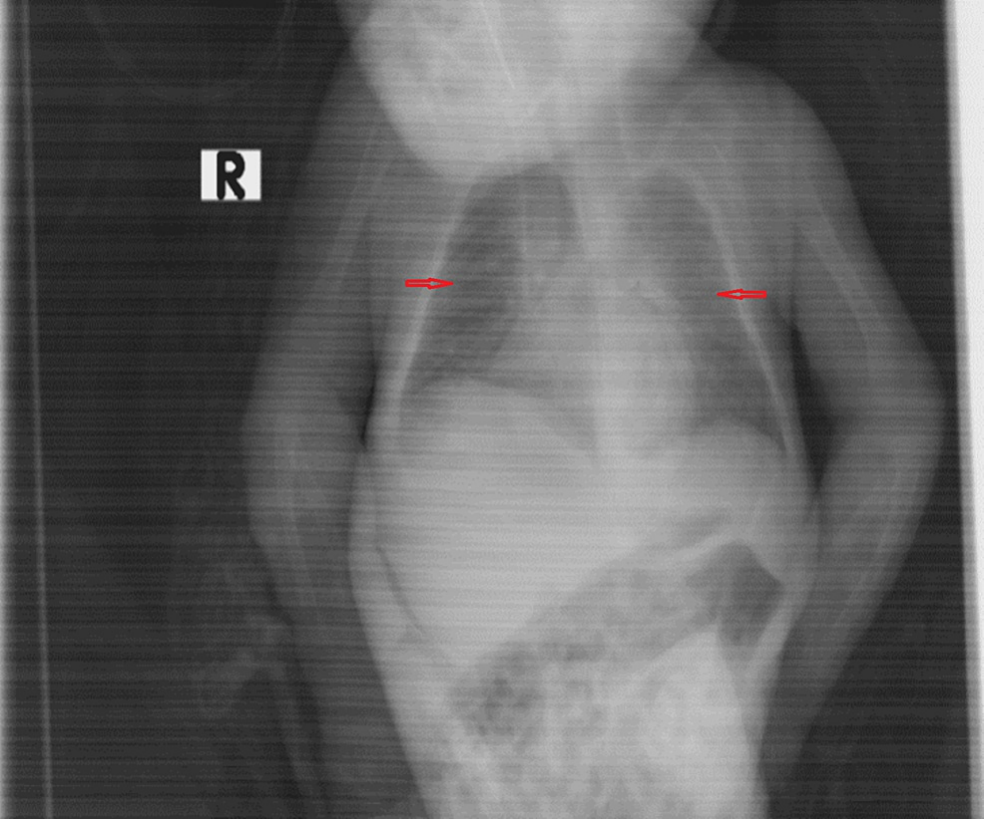
Chest X-rays prior to discharge (normal lungs) This is a radiograph showing both lung fields appearing symmetrical and clear without any visible abnormalities, indicating the restoration of normal lung architecture.

During the recovery phase following extubation, the patient was alternately treated with high-flow nasal cannula (HFNC) therapy and non-invasive positive pressure ventilation (NIPPV) [7], along with continuous respiratory care including bronchodilators, suctioning, and chest physiotherapy. After one week of this treatment, a chest X-ray was repeated and showed bilateral normal lungs, as shown in Figure 4. The child was stable enough to be discharged home with normal laboratory investigations (Table 2).

**Table 2:** Laboratory investigation upon discharge Hgb: hemoglobin; WBC: white blood cell; Na+: sodium; K+: potassium; BUN: blood urea nitrogen; PT: prothrombin time; PTT: partial thromboplastin time; INR: international normalized ratio.

Laboratory investigation	Upon discharge
Hgb	11.4 g/dL
WBC	8.3 × 10^3^/µL
Platelets	373 × 10^3^/µL
Na^+^	136 mmol/l
K^+^	3.8 mmol/l
BUN	2
Creatinine	36
PT	14 s
PTT	46.2 s
INR	1.1
Blood culture	Negative

## Discussion

Infants born with high-risk factors, especially preterm, typically face significantly higher hospitalization rates. This vulnerability becomes more pronounced if they are diagnosed with RSV. Such diagnoses can lead to severe critical illnesses, necessitating intensive medical care [3]. This situation underscores the importance of early detection and proactive management strategies to mitigate the risks associated with RSV in this susceptible infant population. Consequently, healthcare providers must prioritize monitoring and preventive measures for these high-risk groups to reduce the incidence and severity of RSV-related complications.

However, children under two years old and born at 31-36 weeks gestational age (GA) face a higher risk of hospitalization and PICU admission for community-acquired alveolar pneumonia (CAAP) [1-2], especially RSV-associated CAAP, compared to those born after 36 weeks GA. Late-preterm infants have a 50% higher risk of RSV-related CAAP than full-term infants. Also, preterm children, disproportionately represented in PICU admissions for bronchiolitis [3], exceed the general preterm birth rates (4.4-14.4% globally) and face a greater likelihood of requiring mechanical ventilation than full-term infants. In this pediatric case, the patient admitted to the PICU underwent comprehensive monitoring and treatment due to a severe respiratory condition.

Upon admission, laboratory investigations revealed various abnormalities, including an elevated white blood cell count and indications of potential infection or inflammation, but the blood cultures remained negative (Table 1). Maximal mechanical ventilation didn't help; the child's deteriorating condition included oxygen fluctuations, acidosis, hypoxia, and a left pneumothorax treated with needle decompression and an intercostal tube.

Faced with these challenges, the medical team transitioned the patient to HFOV and commenced treatment with iNO at 40 PPM. This combination of therapies led to a notable improvement in the child’s clinical condition and oxygen levels, and echocardiography showed no signs of pulmonary hypertension. Over a five-day period on HFOV with settings gradually increasing to a maximum iNO of 55 PPM, the patient’s condition began to stabilize [5-6].

Following this phase of intensive treatment, the medical team successfully weaned the patient from HFOV to a conventional ventilator over three days, culminating in a successful extubation. After extubation, the patient was managed with alternating HFNC therapy and NIPPV [7]. In addition to frequent respiratory care, including bronchodilators, suctioning, and chest physiotherapy, after one week of recovery, the infant was discharged home in a stable condition.

Moreover, respiratory failure following extubation often leads to a worse prognosis, marked by a higher incidence of ventilator-associated pneumonia and extended stays in both the ICU and hospital. This particular case demonstrates the effectiveness of HFNC therapy and non-invasive mechanical ventilation in enhancing extubation outcomes in pediatric patients.

This case exemplifies the complexity and challenges of managing severe pediatric respiratory conditions. It highlights the efficacy of advanced ventilation strategies and tailored respiratory therapies in critically ill pediatric patients, underscoring the importance of flexibility and adaptability in treatment approaches in the PICU.

## Conclusions

This case illustrates the challenges and underscores the difficulties of managing severe RSV infection in preterm infants and the potential for recovery with advanced respiratory support techniques and timely interventions. Despite the initial critical presentation and complex course, the patient showed remarkable improvement and was discharged home, underscoring the efficacy of individualized intensive care management in the PICU.
